# Association between HPV-16 and HPV-18 viral loads and severity of cervical pre-invasive lesions in women with and without HIV in Botswana

**DOI:** 10.3389/fonc.2026.1809196

**Published:** 2026-06-23

**Authors:** Leabaneng Tawe, Pleasure Ramatlho, Siqi Zhang, Zackary Salem-Bango, Rebecca Ketlametswe, Daniel P. Morse, Doreen Ramogola-Masire, Erle S. Robertson, Giacomo M. Paganotti, Surbhi Grover

**Affiliations:** 1 Botswana-University of Pennsylvania Partnership, Gaborone, Botswana; 2 School of Allied Health Professions, University of Botswana, Gaborone, Botswana; 3 Biostatistics, University of Pennsylvania, Philadelphia, PA, United States; 4 David Geffen School of Medicine, University of California, Los Angeles, Los Angeles, CA, United States; 5 Department of Chemistry, United States Naval Academy, Annapolis, MD, United States; 6 Department of Obstetrics and Gynecology, Faculty of Medicine, University of Botswana, Gaborone, Botswana; 7 Department of Otorhinolaryngology - Head and Neck Surgery, The Tumor Virology Program, Abramson Comprehensive Cancer Center, Perelman School of Medicine, University of Pennsylvania, Philadelphia, PA, United States; 8 Division of Infectious Diseases, Perelman School of Medicine, University of Pennsylvania, Philadelphia, PA, United States; 9 Department of Radiation Oncology, Perelman School of Medicine, University of Pennsylvania, Philadelphia, PA, United States

**Keywords:** Botswana, cervical intraepithelial neoplasia, HIV, HPV-16, HPV-18, viral load

## Abstract

**Background:**

Cervical cancer is an important global health problem, including in Botswana. Human papillomavirus (HPV) and human immunodeficiency virus (HIV) are responsible for a considerable burden of the disease globally. The significance of HPV viral load in the detection of pre-invasive cervical lesions in patients with and without HIV is still controversial. The present study aimed to explore the correlation between HPV-16 and HPV-18 genotype viral loads and the severity of cervical lesions in pre-cervical cancer patients with and without HIV in Botswana.

**Methods:**

A cross-sectional convenience sampling was adopted using 109 archived residual tissue blocks collected from pre-cervical cancer patients in Gaborone, Botswana, during 2017-2019. Viral load for HPV-16 and HPV-18 has been quantified in pre-invasive cervical lesions using quantitative PCR to evaluate its potential as a predictor of disease severity, and examined correlations with HIV status and pre-cancer stage.

**Results:**

Of the 109 patients, HPV-16 was detected in 33.94% and HPV-18 in 14.68%. Only 3 (2.75%) patients were co-infected with both genotypes, while the remaining patients 56 (48.6%) tested negative for both genotypes. In this study 70.6% of the 109 samples were infected with HIV while 29.4% were HIV-uninfected. For HPV-16, higher cervical intraepithelial neoplasia (CIN) grade was associated with increased HPV viral load, while HPV-18 exhibited a more variable pattern, with the highest HPV viral load observed in CIN II. The presence of HPV-16 and elevated HPV-16 viral load were significant risk factors for CIN III. In contrast, the presence of HPV-18 and higher HPV-18 viral load were associated with increased risk for CIN II, but did not have a significant impact on the risk for CIN III. HIV status did not have a significant impact on viral load of these HPV genotypes.

**Conclusions:**

Higher HPV-16 viral loads are linked to more severe cervical lesions while HPV-18 is more associated with CIN II than CIN III. However, HIV status was not associated with higher viral loads. Larger studies are needed to confirm these findings and improve screening.

## 1 Introduction

Human papillomavirus (HPV) infection is recognized as the principal etiological factor in the development of cervical cancer, one of the leading causes of cancer-related mortality among women worldwide (1, 2). Among the numerous HPV genotypes, HPV-16 and HPV-18 have been identified as the most prevalent high-risk types associated with the progression of cervical lesions to invasive carcinoma (3, 4) at a global level, including Botswana. The severity of cervical lesions, ranging from low-grade dysplasia to high-grade squamous intraepithelial lesions or worse, has been shown to be closely linked to the viral load of these genotypes in some studies (5, 6), but this association needs to be confirmed. Understanding the correlation between HPV-16 and HPV-18 genotype viral loads in the cervical epithelium and the progression of cervical lesions is crucial for early detection and management of cervical cancer precursors, especially in populations with a high prevalence of HIV infection like Botswana. Despite existing research on HPV-related cervical lesions, there remains a gap in understanding if and how viral loads of specific HPV genotypes correlate with lesion severity in pre-cervical cancer patients, particularly concerning the influence of HIV co-infection. Synergistic interplay between the two viruses needs further evaluation. By elucidating the relationship between HPV viral loads and lesion severity in the context of HIV co-infection, this research endeavors to provide valuable insights into the pathogenesis of HPV-associated cervical lesions and guide more targeted interventions for high-risk populations.

In Botswana, over 250, 000 women are in the age group 30–49 years (about a quarter of total female population), and are at high risk of developing cervical cancer (7). Among this subset of women, the HIV prevalence is high. The number of women at-risk of cervical cancer will continue to grow until effective primary prevention efforts are established. While previous studies have established a clear association between HPV infection and cervical carcinogenesis, the relationship between HPV viral load and lesion severity, particularly in pre-cervical cancer patients, remains an area of active investigation (6). The progression from HPV infection to cervical cancer is a multifaceted process influenced by various factors, including viral genotype, viral load, host immune response and epigenetics, co-infections and environmental co-factors (8–10). Among these factors, HPV viral load, defined as the number of viral DNA copies per cell or per sample volume, has emerged as a possible predictor of disease progression (8, 11–15). High viral loads of HPV-16 and HPV-18 have been suggested to be a suitable marker of increased risk of developing high-grade cervical lesions and invasive carcinoma (16–20). Furthermore, the detection and quantification of HPV viral loads have become increasingly feasible with advancements in molecular techniques, such as quantitative real-time PCR (qPCR) (21, 22), and it also applied to formalin-fixed paraffin embedded (FFPE) tissues (23–25). These methods allow for the precise measurement of HPV DNA in cervical specimens, enabling researchers to investigate the relationship between viral load and disease outcome with greater accuracy. Despite the wealth of evidence supporting the role of HPV-16 and HPV-18 genotype viral loads in cervical carcinogenesis, there is still need for further research, particularly focusing on pre-cervical cancer patients’ tissues. Therefore, this study aims to explore the correlation between HPV-16 and HPV-18 genotype viral loads and the severity of cervical lesions in pre-cervical cancer patients with and without HIV in Botswana. We seek to elucidate the relationship between viral load levels and histopathological findings. Such insights may ultimately contribute to the development of more effective screening programs and personalized treatment approaches for individuals at risk of cervical cancer.

## 2 Materials and methods

### 2.1 Study design and clinical specimen collection

This was a cross-sectional study that used a total of 109 archived residual FFPE samples. They were collected in a convenience sampling approach from pre-cervical cancer patients who attended public health facilities for diagnostic and treatment services in the southern part of Botswana, Gaborone, during 2017-2019 and were enrolled in the Ipabalele study (26). The study utilized all readily accessible samples that contained sufficient tissue material for analysis. All tissue samples included in this study were those that were diagnosed as pre-invasive cervical cancer. However, “other” represents benign, reactive, or non-dysplastic histological findings confirmed via biopsy: Normal tissue/HPV effect, cervicitis/Inflammation, and squamous metaplasia. Therefore, although these specific histological findings were identified, all participants within this category had histologically confirmed pre-invasive cervical lesions and fulfilled the study inclusion criteria.

The patients underwent routine cervical cancer screening, and those with cervical abnormalities had a biopsy or tissue removal (i.e. loop electrosurgical excision procedure or LEEP) as part of the screening or diagnostic process. Additionally, enrolment was open to any eligible woman seeking care at a health facility, accompanying someone who was seeking care, or working at or near the facility [26]. Histopathological diagnosis was made by the anatomical pathologist at the Botswana’s Ministry of Health and Wellness National Health Laboratory, a reference laboratory providing pathology services to southern Botswana. Sections of the tissue blocks were analyzed for the histopathological diagnosis of pre-cervical lesions. Pre-cervical lesions were identified through the national cervical screening algorithm using Pap smear cytology results, where samples are classified according to the Bethesda reporting system as NILM (Negative for Intraepithelial Lesion or Malignancy), ASCUS (Atypical Squamous Cells of Undetermined Significance), ASC-H (Atypical Squamous Cells–cannot exclude High-grade Squamous Intraepithelial Lesion), LSIL (Low-grade Squamous Intraepithelial Lesion), HSIL (High-grade Squamous Intraepithelial Lesion), AGC (Atypical Glandular Cells), AGC-FN (Atypical Glandular Cells–Favor Neoplastic), and AGC-AIS (Atypical Glandular Cells–Adenocarcinoma *In Situ*), with abnormal findings prompting further clinical evaluation and histopathological confirmation of pre-invasive lesions. Baseline demographic and clinical data including HIV status, histopathology result, and year of diagnosis were obtained from the Integrated Patient Management System (IPMS) and patient charts.

### 2.2 Tissue sectioning

Prior to sectioning, the paraffin-embedded tissue blocks were placed on ice to cool down and ensure the tissue remained firm. The tissue sections were then carefully cut using a semiautomatic precision microtome (Slee CUT 5062, Nieder-Olm, Germany), with the clearance angle set according to the manufacturer’s specific instructions. To maintain consistency and cleanliness, the first 20-μm tissue section from each block was discarded, as this initial cut could contain impurities or irregularities, ensuring that the subsequent sections were cleaner and more suitable for analysis. Strict aseptic techniques were rigorously followed when handling the tissue blocks to avoid contamination. All samples were transferred using sterile instruments, minimizing any risk of introducing foreign material. After each tissue block sectioning, the microtome stage and blade were thoroughly cleaned with xylene to remove any paraffin residue, followed by a wash with absolute alcohol and DNA/RNA Away solution to eliminate any potential DNA or RNA contaminants. To further safeguard against cross-contamination between different tissue samples, blank paraffin control blocks were sectioned after every 10th sample, and these control blocks were subsequently analyzed for the presence of HPV DNA to verify that no unintended transfer had occurred between sections.

### 2.3 DNA extraction and quantification

DNA was extracted from the archived FFPE tissue using the AllPrep DNA/RNA FFPE Kit (Qiagen, Hilden, Germany) according to the manufacturer’s instructions. The extracted DNA was then quantified, and its purity assessed using Qubit Fluorometric before analysis. Most of the samples were above 60 ng/ul DNA concentration, with some inter-sample variation that however, did not impact on PCR efficiency.

### 2.4 Typing and estimation of HPV-16 and HPV-18 viral load

Two primers’ pairs and probes were used to individually detect HPV genotypes 16 and 18, according to an existing protocol for E7 gene (23), as these two genotypes are the most commonly reported and considered high risk strains linked to the development of cervical and other cancers.

HPV DNA amplification was carried out using a Real-Time quantitative TaqMan PCR assays. HPV viral load was estimated using a technique similar to Manake et al. (27) with adaptations that followed guidelines of Pfaffl (28). In short, standard curves for absolute quantification were derived from positive HPV plasmid amplicons of the target region. Gene copy number per microliter of the DNA extract within a specific positive control sample was estimated as the concentration of that particular sample, divided by the molecular weight of the target region, multiplied by Avogadro’s number. Serial dilutions of the aforementioned positive control were performed with accompanying qPCR to generate a best fit linear regression model. Cycle threshold (Ct) values from processed samples were inputted into this model to generate an estimated viral load. Viral load was finally expressed as base-10 logarithm of the HPV copy numbers per microliter. Human betaglobin gene amplification and quantification was carried out in a similar manner on samples nucleic acid extracts to determine sample’s cellularity and normalize HPV viral loads to tissue quantity. The thermal cycling conditions, optimized to obtain the best amplification kinetics under the same temperatures and composition of reaction mixture, consisted of the following thermal profile: 2 minutes at 50 °C, 10 minutes at 95 °C and 40 cycles of 15 seconds at 95 °C and 1 minute at 60 °C.

### 2.5 Statistical analysis

We evaluated the distribution of HPV log viral load data for normality using Shapiro-Wilk test and compared HPV mean viral load by HIV status for the two genotypes using Wilcoxon rank-sum test, a non-parametric test suitable for data where normality assumption cannot be met, as was the case in our study. The distribution of categorical variables between HPV genotypes was compared by χ^2^ test. Multivariate logistic Regression analysis was performed to evaluate which factors potentially influencing the likelihood of having CIN III/micro-invasion versus CIN I/II. Multivariate logistic Regression analysis was performed to evaluate which factors potentially influencing the likelihood of having CIN III/micro-invasion versus CIN I/II. Variables were selected for multivariate model based on known clinical relevance or association threshold of p < 0.2 in univariate analysis. Data cleaning and preparation were conducted in R (Version 4.2.3).

## 3 Results

### 3.1 Demographic and clinical characteristics of study subjects

Clinical and demographic characteristics of study subjects were retrieved from the IPMS, used in the public health of Botswana. They are summarized in **Table 1**. All available tissue samples from women with a confirmed diagnosis of pre-cervical cancer were included. A total of 109 FFPE tissue blocks with colposcopy-detectable cervical lesion were included in this study. Out of 109 specimens, 77 (70.6%) were from HIV-infected women and 32 (29.4%) were from HIV-uninfected women. Median age of the study samples was 38 years (IQR = 12.0). The mean age of HIV-positive patients was 1.1 years less than that of HIV-negative (*p* = 0.625). The distribution of cases included: 5 (4.6%) with histological diagnosis of CIN I, 12 (11.0%) CIN II, 76 (69.7%) CIN III, 3 (2.8%) micro-invasion and 13 (11.9%) had “other” clinical manifestations. This last category refers to symptoms or signs that are not typically associated with the primary or most common indicators of cervical cancer. These manifestations can include a variety of less common symptoms, which may be related to advanced stages of the disease or to other underlying conditions.

**Table 1:** Demographic and clinical characteristics of patients (n = 109).

Variable	Overall
Age	
Mean (+/-SD)	39.56 (9.43)
Median (IQR)	38.00 (12.00)
Min, Max	21.00, 65.00
HIV status	
Positive N (%)	77 (70.64%)
Negative N (%)	32 (29.36%)
Marital status	
Married N (%)	17 (15.60%)
Single N (%)	86 (78.90%)
Divorced N (%)	3 (2.75%)
Widowed N (%)	3 (2.75%)
Ever screened for cervical cancer	
Yes N (%)	107 (98.17%)
No N (%)	2 (1.83%)

IQR, interquartile range; HIV, human immunodeficiency virus; SD, standard deviation.

### 3.2 Distribution of HPV-16 and -18 genotypes and viral load

Out of the 109 patients, 37 (33.9%) were positive for HPV-16, 16 (14.7%) for HPV-18, including 3 (2.8% out of the total samples, corresponding to 5.7% of the HPV-16 and HPV-18 positives) positive for both HPV-16 and HPV-18, the remaining 56 samples (48.6%) were negative for both HPV-16 and HPV-18. Among patients diagnosed with CIN I (N = 5), HPV-16 infection was observed in 1 patient (20%), while HPV-18 infection was present in 3 patients (60%). In CIN II (N = 12), 5 patients (41.7%) had HPV-16 and 2 patients (16.7%) had HPV-18. For CIN III (N = 76), HPV-16 was detected in 28 patients (36.8%) and HPV-18 in 9 patients (11.8%). In cases of micro-invasion, HPV-18 was found in 1 patient (33.3%). Among the “other clinical manifestation” category, HPV-16 was present in 3 patients (23.1%), with HPV-18 detected in 1 patient (7.7%). For the data breakdown and viral load according to the HPV genotype, see **Table 2**. The prevalence of HPV-16 seems to increase with higher CIN grades, particularly in CIN III, and the viral load was higher in CIN III compared to CIN I and CIN II. In contrast, HPV-18 exhibited notable prevalence in CIN I and the highest viral load was observed in CIN II, which contrasts with HPV-16 trends (see **Table 2**).

**Table 2:** Distribution of grade of cervical lesions according to the HPV genotype, and viral load.

HPV-16 positive (N = 37)	N (%)	Viral load log (+/-SD)
CIN I	1 (2.7)	3.15 (NA)
CIN II	5 (13.5)	3.89 (0.99)
CIN III	28 (75.7)	4.97 (1.87)
Micro Invasion	0 (0.0)	NA
Other ‡	3 (8.1)	4.00 (0.18)
HPV-18 positive (N = 16)		
CIN I	3 (18.8)	6.25 (0.83)
CIN II	2 (12.5)	4.97 (0.55)
CIN III	9 (56.3)	7.26 (3.70)
Micro Invasion	1 (6.3)	4.55 (NA)
Other ‡	1 (6.3)	6.02 (NA)

CIN, cervical intraepithelial neoplasia; HPV, human papillomavirus; NA, not applicable.

‡, benign, reactive, or non-dysplastic histological findings confirmed via biopsy: Normal tissue / HPV effect, cervicitis / Inflammation, and squamous metaplasia.

### 3.3 Comparison of the viral load for HPV-16 and HPV-18 by HIV status

There was no statistically significant difference in HPV-16 and HPV-18 viral load between HIV-positive and HIV-negative individuals (*P*-value = 0.858 and *P*-value = 0.491, respectively) (see **Table 3**). Shapiro-Wilk test is applied to test the normality of the viral load data, confirming that both HPV 16 (p < 0.001) and HPV 18 (p < 0.001) significantly deviated from a normal distribution.

**Table 3:** Comparison of viral load for HPV-16 and HPV-18 by HIV status.

HPV genotype (N)	HIV status, N (%)	Viral load mean (+/-SD)	*P* - value
HPV-16 (37)	Positive, 25 (67.6%)	5.03 (2.28)	0.858
	Negative, 12 (32.4%)	4.45 (1.62)	
HPV-18 (16)	Positive, 9 (56.2%)	6.30 (2.71)	0.491
	Negative, 7 (43.7%)	6.78 (3.21)	

HIV, human immunodeficiency virus; SD, standard deviation. The *P-*value is associated to the Wilcoxon rank-sum test.

### 3.4 Clinical and virological differences between patients with CIN I/CIN II vs CIN III/micro-invasion

There was no statistically significant difference in the presence of HPV-16 and HPV-18 between CIN I/CIN II and CIN III/micro invasion (see **Table 4**). However, there was a significant difference in HPV-16 viral load (*P*-value = 0.035), indicating that individuals with CIN III/micro-invasion had higher viral load compared to those with CIN I/CIN II. Instead, HPV-18 viral load was not associated (*P*-value = 0.322). Regarding HIV status, there was no difference in HIV status between these CIN groups (*P*-value = 0.165). Among HIV-positive individuals, however, there is a statistically significant difference in HPV-16 viral load (*P*-value = 0.004), with higher viral loads observed in those with CIN III/micro-invasion compared to CIN I/CIN II. For HPV-18, no difference in viral load between the two groups was detected (see **Table 4**).

**Table 4:** Comparison of various clinical and virological characteristics between patients with CIN I / CIN II vs CIN III / micro-invasion.

Variable	CIN I / CIN II	CIN III / micro-invasion	*P* - value
Age			
Mean (SD)	35.06 (9.00)	39.51(8.23)	0.074 **
Median (IQR)	35.00 (12.00)	28.00 (11.50)	
Min, Max	21.00, 52.00	23.00, 62.00	
Marital status			
Married, N (%)	1 (5.88%)	12 (15.19%)	0.720***
Single, N (%)	16 (94.12%)	62 (78.48%)	
Divorced, N (%)	0 (0.00%)	3 (3.80%)	
Widowed, N (%)	0 (0.00%)	2 (2.53%)	
Screening history			
Yes, N (%)	16 (94.12%)	78 (98.73%)	0.324***
No, N (%)	1 (5.88%)	1 (1.27%)	
HPV-16			
Positive, N (%)	6 (35.29%)	28 (35.44%)	0.999*
Negative, N (%)	11 (64.71%)	51 (64.56%)	
HPV-18			
Positive, N (%)	5 (29.41%)	10 (12.66%)	0.175*
Negative, N (%)	12 (70.59%)	69 (87.34%)	
Viral load log (HPV-16)			
Mean (SD)	3.76 (0.94)	4.97 (1.87)	0.035**
Median (IQR)	3.50 (0.89)	4.04 (2.22)	
Min, Max	3.02, 5.46	2.98, 9.37	
Viral load (HPV-18)			
Mean (SD)	5.73 (0.95)	6.99 (3.59)	0.322**
Median (IQR)	5.35 (1.04)	5.86 (1.52)	0.999*
Min, Max	4.58, 6.99	4.49, 13.78	
HIV status			
Positive, N (%)	10 (58.82%)	62 (78.48%)	0.165*
Negative, N (%)	7 (41.18%)	17 (21.52%)	
HPV-16 viral load among HIV positive			
Mean (SD)	3.49 (0.52)	5.06 (1.84)	0.004**
Median (IQR)	3.46 (0.84)	4.06 (2.54)	
Min, Max	3.02, 4.02	2.98, 9.37	
HPV-16 viral load among HIV negative			
Mean (SD)	4.30 (1.64)	4.70 (2.06)	0.803**
Median (IQR)	4.30 (1.16)	3.77 (0.94)	
Min, Max	3.15, 5.46	3.09, 9.12	
HPV-18 viral load among HIV positive			
Mean (SD)	5.87 (0.74)	6.55 (3.51)	0.671**
Median (IQR)	5.87 (0.52)	5.43 (1.24)	
Min, Max	5.35, 6.39	4.49, 13.60	
**HPV-18 viral load HIV negative**			0.417**
Mean (SD)	5.64 (1.23)	7.65 (4.16)	
Median (IQR)	5.35 (1.21)	6.13 (2.47)	
Min, Max	4.58, 6.99	4.55, 13.80	

HIV, human immunodeficiency virus; HPV, human papillomavirus; CIN, cervical intraepithelial neoplasia; SD, standard deviation, IQR, interquartile range.

* = Chi-square test associated P-value; ** = Student's t-test associated P-value.

*** fisher’s exact test.

### 3.5 Factors associated with CIN II and CIN III

The study found no significant association between disease outcome (CIN I/II and CIN III/micro-invasion) and age, viral loads, absence of HPV-16 and -18 genotypes on multivariate logistic regression analysis (**Table 5**).

**Table 5:** Factors associated with the severity of cervical pre-invasive lesions in women with and without HIV in Botswana.

Variable	OR (95% CI)	*P* - value
Age*	1.07 (1.00- 1.16)	0.064
HPV-16 (negative)**	17.46 (0.67-3391.20)	0.157
HPV-16 viral load log*	1.39 (0.91-7.61)	0.223
HPV-18 (negative)**	13.03 (0.39- 4059.75)	0.208
HPV-18 viral load log*	1.34 (0.81-3.71)	0.371
HIV (negative)**	0.42 (0.12-1.44)	0.156

HIV, human immunodeficiency virus; HPV, human papillomavirus; CIN, cervical intraepithelial neoplasia; OR, odds ratio.

*Continuous variables.

**Categorical variable.

## 4 Discussion

In this study we investigated the possible association between HPV viral load (for HPV -16 and HPV-18 genotypes) and the severity of the pre-cancer cervical lesion in patients with or without HIV from Botswana. Results indicate that there was a statistical significant association observed in the viral load for HPV-16 but not HPV-18 across different grades of CIN. Among HIV-positive subjects, HPV-16 (but not HPV-18) showed a statistically significant increase in mean viral load according to the severity of lesions. The results may help to understand how the viral density in the cervix epithelium associates with the risk of cervical disease progression. Based on the result of this study it is possible to speculate that HPV-16 viral load may be clinically useful in predicting cervical dysplasia especially in women living with HIV.

Several studies have reported the close relationship between several high-risk HPV genotypes and the severity of cervical lesions, as well as treatment prognosis (29–31). Similarly, to Xi et al. (32), Berggrund et al. (33) demonstrated that high-risk HPV viral load was linked with CIN II and above lesions. Also, Dong et al. (34) reported that HPV-16/31/33/52/58 viral load correlated with cervical lesion severity. Conversely, Del Rio-Ospina et al. (35) suggested that a low HPV-16 viral load exhibited a stronger association with cervical lesions than a high HPV-16 load. Shen et al. (36) and Long et al. (37) also reported associations between high-risk HPV viral load and cervical lesion severity, specifically highlighting the relevance of HPV-16/33/58 viral load with premalignant cervical lesions. HPV-18 exhibited a higher mean and median viral load compared to HPV-16. This contrasts with the findings of Obeid et al. (23), who reported different results, indicating that HPV-16 had a higher median viral load than HPV-18 overall. Additionally, while some studies have reported a linear correlation between HPV-16 viral load and cervical abnormalities (34), this trend was not observed for HPV-18, consistent with the findings of the present study. Furthermore, no difference in mean viral load between HIV positive and negative women was detected for HPV-16 and HPV-18, as reported in other studies (38, 39). However, among subjects with HIV infection, HPV-16 viral load showed a significantly positive association with the severity of cervical lesions, what has not been observed in this cohort for HPV-18. Previous studies, including those conducted in Botswana, have demonstrated a positive correlation between HIV infection and the presence of the HPV-16 genotype. However, this association does not extend to HPV-18 or other high-risk HPV (40, 41).

The HPV genotypes identified in this study comprised of HPV-16 (33.9%) and HPV-18 (14.7%). These findings align with those of Macleod et al. (42), who reported HPV-16 prevalence at 26% and HPV-18 prevalence at 16.8% among HIV-positive patients in Botswana. Similarly, a study by Ramogola-Masire et al. (43) observed HPV-16 prevalence at 45% and HPV-18 prevalence at 26%, focusing solely on HIV-positive patients in Botswana. Discrepancies in prevalence rates between these studies may be due to differences in HPV genotype detection methods, and patient populations. In summary, the findings suggest that HPV-16 prevalence and viral load seems to increase with increasing CIN grades, whereas HPV-18 shows a more variable pattern, including the highest viral load in CIN II, that we cannot exclude as an effect of small sample size. There are several limitations to the present analysis. Firstly, the patients had only one time point, and the sample size was small, but still very representative of the total population of 2, 346, 179 (44). Extended follow-up and study of greater numbers of patients may be required before a definite conclusion about the prognostic value of HPV-viral load. Another limitation is that the study only focused on HPV genotypes -16 and -18. While the method demonstrated high specificity for these two genotypes, it does not account for other high-risk or low-risk HPV genotypes that may also contribute to the development of cervical or other HPV-associated cancers.

## 5 Conclusion

This study highlights the association between HPV-16 viral load and the severity of cervical lesions, with higher viral loads linked to more advanced lesions, regardless of HIV status HPV-16 is the predominant genotype in CIN II lesions, while HPV-18, although less prevalent, exhibited a higher mean viral load. HIV infection significantly correlates with HPV-16 viral load but not HPV-18. These findings suggest that monitoring HPV-16 viral load, particularly in HIV-positive individuals, may help identify those at greater risk for severe cervical lesions. However, the small sample size limits the robustness of these conclusions, and future research with larger sample sizes is needed to confirm these results and refine screening and intervention strategies.

## Data Availability

The raw data supporting the conclusions of this article will be made available by the authors, without undue reservation.
